# Supplementary material for: Gene silencing by double-stranded RNA from C. elegans neurons reveals functional mosaicism of RNA interference
Source: Nucleic Acids Res. 2019 Sep 10;47(19):10059–71. doi: 10.1093/nar/gkz748 (PMC6821342; doi:10.1093/nar/gkz748)
Supplement: gkz748_Supplemental_Files [file gkz748_supplemental_files.zip › Ravikumar_et_al_2019_Supplemental_Submission.pdf]

SUPPLEMENTARY DATA

SUPPLEMENTARY FIGURES AND FIGURE LEGENDS

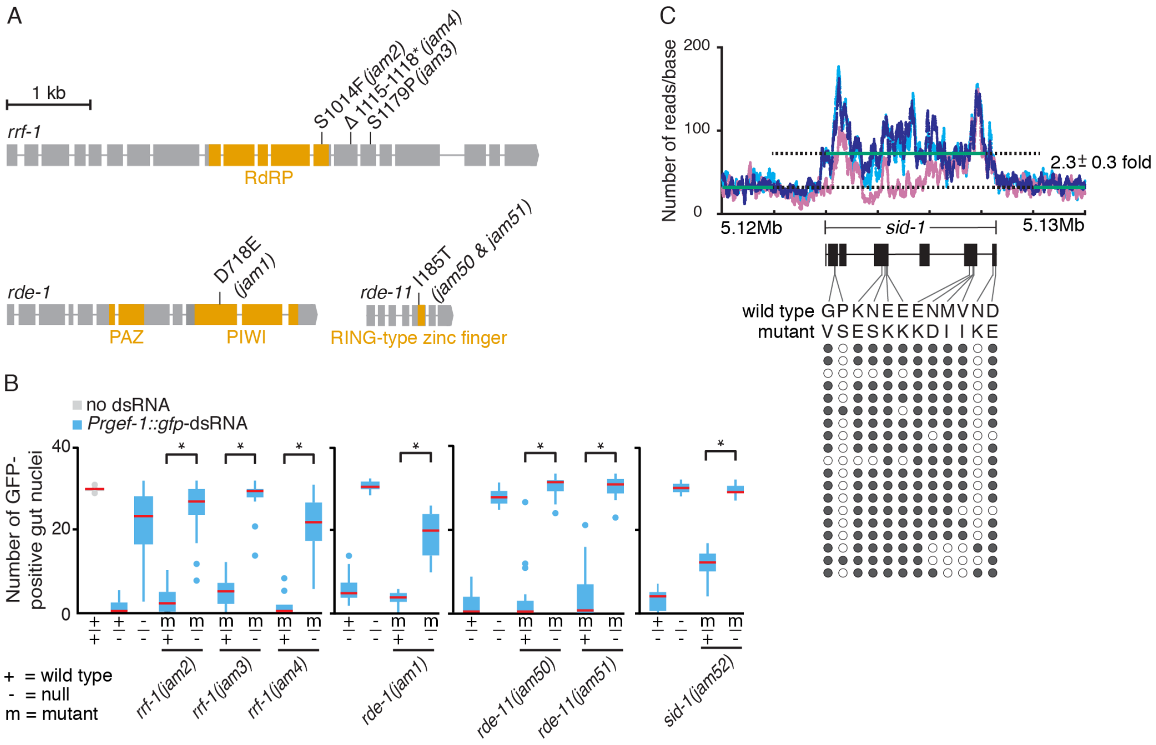

Supplementary Figure S1: Four genes with known roles in RNAi were identified among mutants defective in silencing upon *eri-1* loss and/or by neuronal dsRNA. (**A**) Schematic of *rrf-1*, *rde-1*, and *rde-11* genes with identified molecular lesions. The gene structures (exons = boxes, introns = lines), identified alleles along with deduced changes in the corresponding proteins (*jam2*(S1014F), *jam3*(S1179P), and *jam4(*Δ1115-1118*, short insertion/deletion resulting in downstream stop codon) in *rrf-1*, *jam1*(D718E) in *rde-1*, and *jam50*(I185T) and *jam51*(I185T) in *rde-11*), and regions encoding known protein domains (in orange, RdRP in *rrf-1*, PAZ and PIWI in *rde-1*, and RING-type zinc finger in *rde-11*) are indicated. Scale bar = 1 kb. (**B**) Each isolated allele fails to complement its corresponding null allele. Silencing in test crosses was scored by counting the number of GFP-positive intestinal nuclei in male cross progeny (m/-) when mutant animals (m) were each crossed to animals with a null allele *(-).* Male cross progeny of mutants crossed with N2 (m/+) and

of the null mutant crossed to the strain used for mutagenesis (AMJ1, see strain list for complete genotype) (+/-) were scored as controls. Red bar, n and asterisks are as in Figure 1C. Unlike in *rde-1* null mutants, silencing is detectable in *rde-1(jam1)* animals, indicating that the *jam1* mutation results in a partial loss of function. Both *jam50* and *jam51* mapped to chromosome IV and an examination of all mutations identified by whole genome sequencing revealed identical mutations in *rde-11* and in other genes, suggesting that these mutants are siblings. Sequencing of the exons of *sid-1* in animals with *jam52* did not reveal any mutations although *jam52* maps to chromosome V and complements *rde-1* but not *sid-1*. (**C**) One of the transgenes present in AMJ1 includes a mutated copy of *sid-1* gene sequence incorporated from a PCR fragment. Illumina sequencing reads covering each base for three different mutants (cyan, navy, magenta) indicate that the average coverage (green lines) within the *sid-1* gene is ~2 fold higher than that in surrounding regions, consistent with the presence of one additional copy in the background. Twelve changes in the *sid-1* gene (in >15% of reads covering a base) that altered the encoded amino acid (wild-type and mutant residues are indicated) were detected in at least 2 of 19 sequenced mutants (filled circles). Consistently, this mutated copy of *sid-1*, which is a part of the *qtIs50* transgene, is non-functional (95.3% silencing of *dpy-7* in *qtIs50* animals versus 1.7% silencing in *sid-1(-); qtIs50* animals by feeding RNAi, n>40 L4-staged animals).

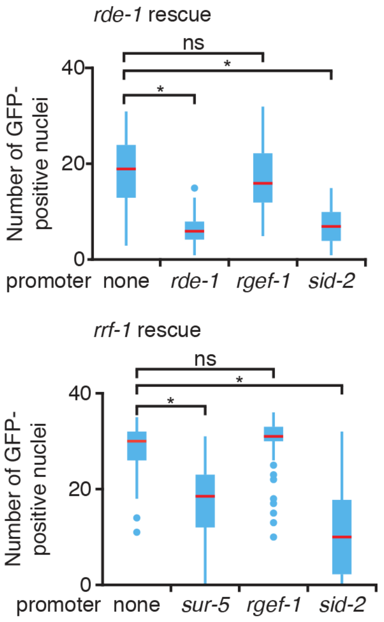

Supplementary Figure S2: *rde-1* and *rrf-1* are required in the intestine for silencing by neuronal dsRNA. *Top*, Rescue of *rde-1(jam1)* using tissue-specific promoters indicates that RDE-1 functions in intestinal cells but not in neurons to enable silencing by neuronal dsRNA. The number of GFP-positive intestinal nuclei in *rde-1(jam1)* animals that were transformed with either a co-injection marker alone (none) or a co-injection marker along with *rde-1(+)* expressed under its own (*rde-1*), intestine-specific (*sid-2*), or neuron-specific (*rgef-1*) promoter were counted. *Bottom*, Rescue of *rrf-1(jam3)* using tissue-specific promoters indicates that RRF-1 functions in intestinal cells but not in neurons to enable silencing by neuronal dsRNA. The number of GFP-positive intestinal nuclei in *rrf-1(jam3)* animals that were transformed with either a co-injection marker alone (none) or a co-injection marker along with *rrf-1(+)* expressed under a somatic (*sur-5*), intestine-specific (*sid-2*), or neuron-specific (*rgef-1*) promoter were counted. Red bar, n and asterisks are as in Figure 1C, and ns = not significant.

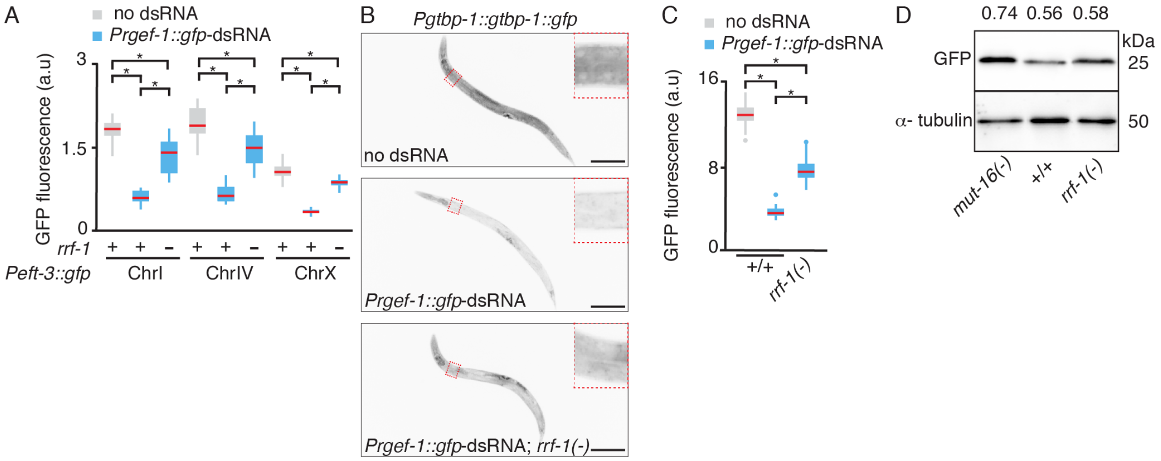

Supplementary Figure S3: Silencing in the absence of RRF-1 can occur at multiple *gfp* targets expressed under the control of different regulatory elements. (**A**) Silencing in the absence of RRF-1 does not depend on chromosomal location of target sequences. Effect of *Prgef-1::gfp*-dsRNA and loss of *rrf-1* on GFP fluorescence in animals with *Peft-3::gfp* transgenes located on different chromosomes was quantified as in Figure 2B. Grey boxes, cyan boxes, red bars, n, and asterisks are as in Figure 2B. (**B-C**) A single-copy gene fusion generated using Cas9-based genome editing can be silenced by neuronal dsRNA in *rrf-1(-)* animals. Representative L4-staged animals that express GFP in all tissues (*Pgtbp-1::gtbp-1::gfp*, *top*) and animals that in addition express *Prgef-1::gfp*-dsRNA in *rrf-1(+)* or *rrf-1(-)* backgrounds (*middle* or *bottom*, respectively) are shown (B). Insets are representative of the region quantified in multiple animals. Quantification of silencing for GFP expressed from *Pgtbp-1::gtbp-1::gfp* is shown (C). Grey boxes, cyan boxes, red bars, n, and asterisks are as in Figure 2B. Scale bar = 50 μm. **(D)** Silencing in the absence of RRF-1 is associated with a detectable decrease in protein levels. Western blot of GFP protein levels in *Peft-3::gfp* animals expressing *gfp*-dsRNA in an otherwise wild type background (+/+), *mut-16(-)* background (no silencing) or *rrf-1(-)* background (partial silencing). Levels of GFP are normalized to α-tubulin and the median ratios of 3 technical replicates are shown.

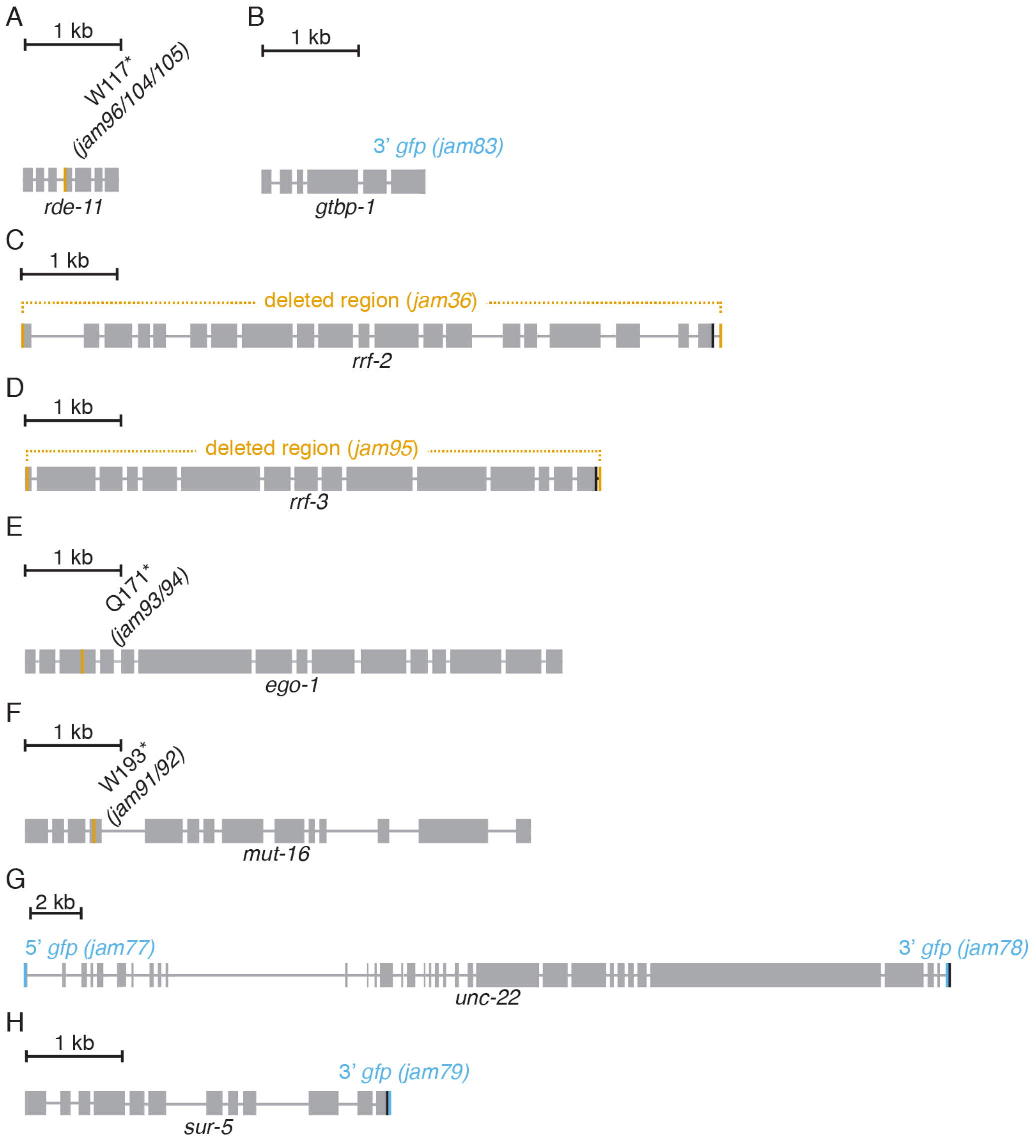

Supplementary Figure S4: Schematic of genomic changes made using Cas9-based genome editing. Genomic changes introduced into the *rde-11*, *gtbp-1*, *rrf-2*, *rrf-3*, *ego-1*, *mut-16*, *unc-22*, and *sur-5* loci in this study are indicated. The sgRNA target site is indicated (blue for insertions and orange for point mutations and deletions) on the gene structure (exons = grey boxes, introns = grey lines, stop codon = black). Homology-directed repair templates were used to either insert *gfp* sequences (**B**, **G** and **H**), create point mutations (**A**, **E** and **F**) or to delete the region between two target sites (**C** and **D**).

Asterisks indicate stop codons or for *ego-1*, disruption of the reading frame, and scale bars are as indicated.

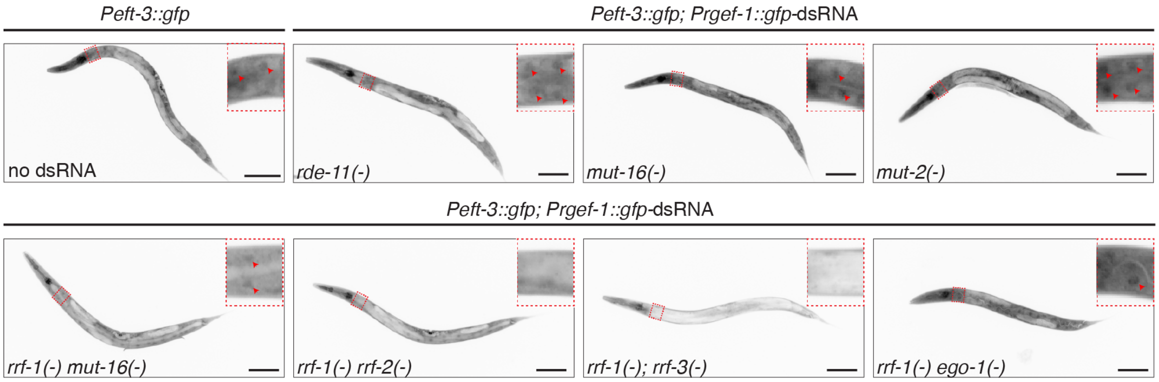

Supplementary Figure S5: Silencing by neuronal dsRNA in the absence of RRF-1 requires EGO-1 and mutator proteins. Representative images of L4-staged animals expressing no dsRNA or expressing *Prgef-1::gfp*-dsRNA in various single and double mutant backgrounds are shown. Persistent fluorescence in the cytoplasm and nucleus (red arrows) is visible in unsilenced animals (*rde-11(-), mut-16(-), mut-2(-), rrf-1(-) mut-16(-)* and *rrf-1(-) ego-1(-)).* GFP fluorescence in insets are quantified in Figure 2B and 2C.

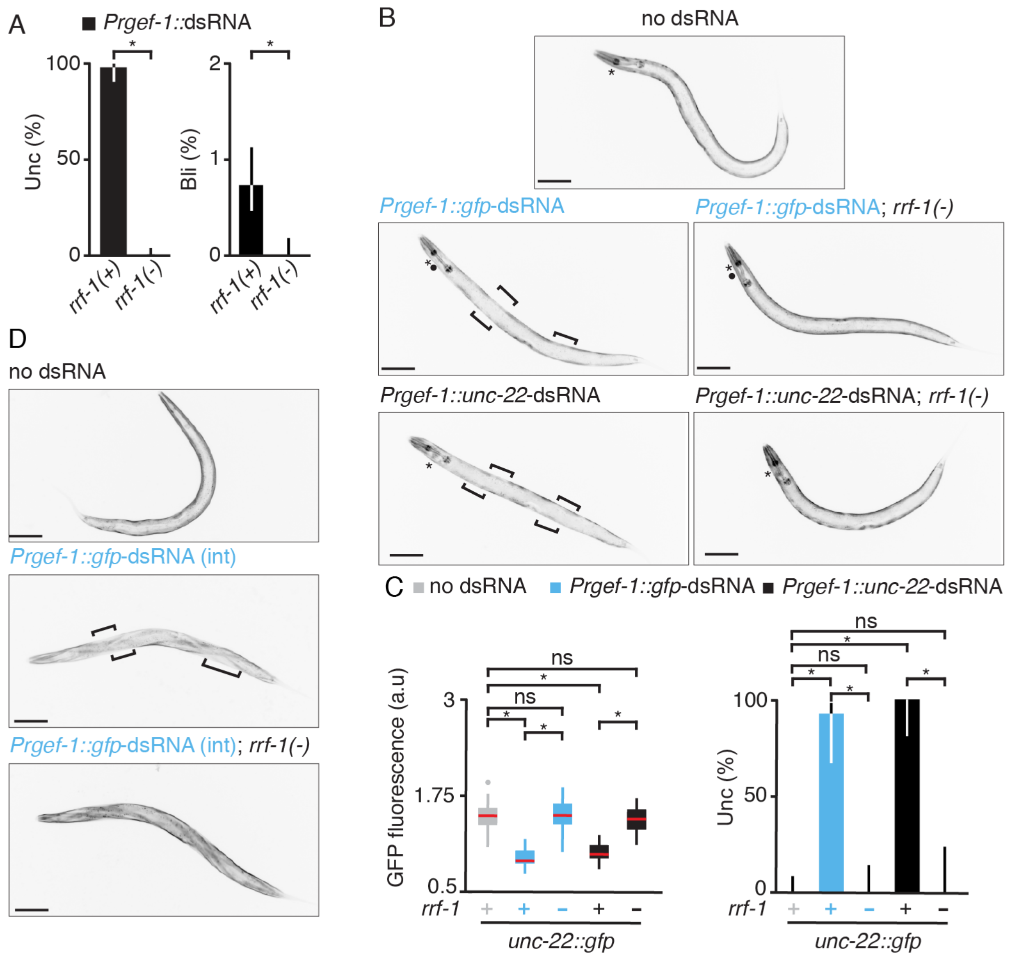

Supplementary Figure S6: Silencing of a chimeric target by two sources of dsRNA suggests target context and not source of dsRNA dictates RRF-1 requirement. (**A**) Silencing of *unc-22* or *bli-1* by neuronal dsRNA requires RRF-1. Percentage of animals showing silencing of *unc-22* or *bli-1* by matching dsRNA expressed under a neuronal promoter (*Prgef-1*) was measured in *rrf-1(+)* or *rrf-1(-)* backgrounds. Asterisks indicate p-value < 0.05 (Wilson's estimates for proportions), n=75 L4-staged animals for *unc-22* and n ≥ 2650 gravid adult animals for *bli-1*. (**B-C**) Effect of *rrf-1* loss on silencing of a single chimeric target *Punc-22::unc-22::gfp* by either *Prgef-1::gfp*-dsRNA or *Prgef-1::unc-22*-dsRNA was characterized as in Figure 3. Fluorescence in the pharynx is observed in some cases because of expression from *Punc-22::unc-22::gfp* (asterisk, See Materials and Methods) or because of fluorescence from co-injection markers (circle, see Figure legend

3A). **(D)** An integrated source of neuronal dsRNA that enables silencing in the absence of RRF-1 similarly changes its requirement when used to silence a different target. Representative L4-staged animals that express GFP from *Punc-22::gfp::unc-22* and animals that in addition express *Prgef-1::gfp*-dsRNA (along with a *rol-6* co-injection marker) integrated into a chromosome (int) in *rrf-1(+)* or *rrf-1(-)* backgrounds (*middle* or *bottom*, respectively) are shown. Scale bar = 50 μm.

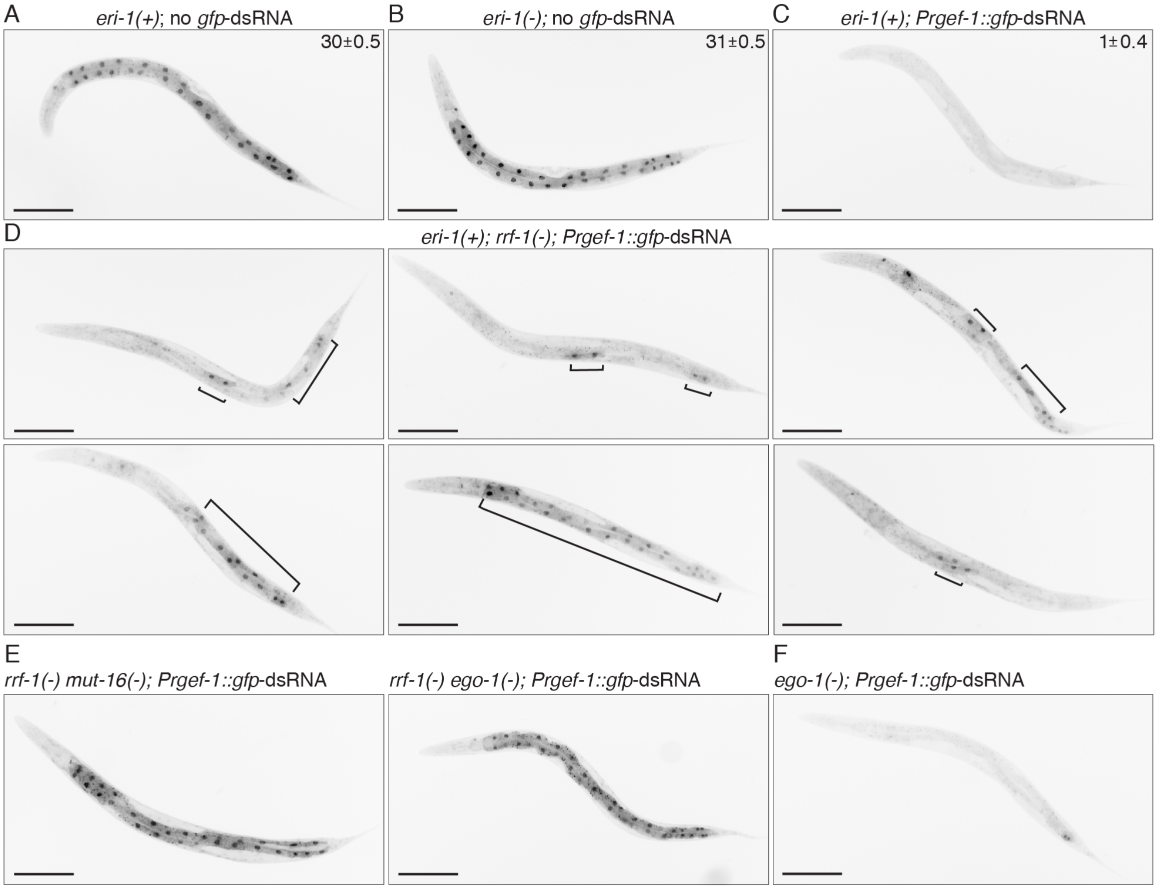

Supplementary Figure S7: Single-copy *sur-5::gfp* is not subject to self-silencing upon *eri-1* loss but can be robustly silenced by neuronal dsRNA, revealing different patterns of MUT-16- and EGO-1-dependent silencing in an *rrf-1(-)* background. (**A-C**) Representative *sur-5::gfp* animals in an *eri-1(+)* (i.e., wild-type) background (A) or an *eri-1(-)* background (B) that do not express *gfp*-dsRNA or in an *eri-1(+)* background that express *Prgef-1::gfp-*dsRNA (C) are shown. Average numbers of GFP-positive intestinal nuclei are indicated, errors indicate 95% confidence intervals and n = 25 L4-staged animals. (**D**) A selection of L4-staged *sur-5::gfp* animals that express *Prgef-1::gfp*-dsRNA in an *eri-1(+); rrf-1(-)* background are shown. Brackets indicate intestinal nuclei that are strongly dependent on RRF-1 for silencing. (**E**) Silencing in the absence of RRF-1 requires MUT-16 and EGO-1. Representative *sur-5::gfp* animals expressing *Prgef-1::gfp*-dsRNA in *rrf-1(-) mut-16(-)* or *rrf-1(-) ego-1(-)* backgrounds are shown. (**F**) EGO-1 is required for silencing in intestinal

cells only in the absence of RRF-1. A representative *sur-5::gfp; ego-1(-)* animal expressing *Prgef-1::gfp*-dsRNA is shown. Scale bar = 50 µm in all panels.

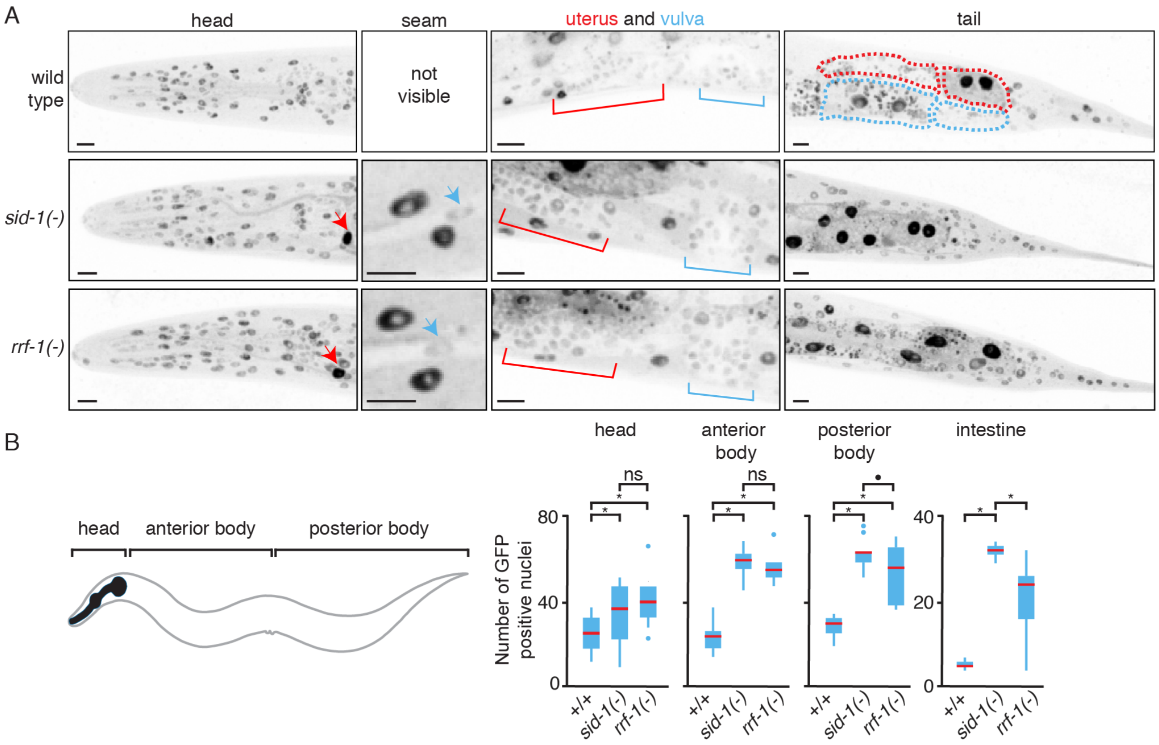

Supplementary Figure S8: A single-cell resolution view of silencing by neuronal dsRNA reveals differences among cell types and a requirement for RRF-1 in most non-intestinal cells. Silencing of individual nuclei was examined using confocal microscopy of *sur-5::gfp* animals that express *Prgef-1::gfp*-dsRNA in a wild-type, *sid-1(-),* or *rrf-1(-)* background. (**A**) Representative images showing maximum intensity projections that highlight extents of silencing in different cell types. The excretory canal cell (red arrow), seam cell (cyan arrow), cells of the developing uterus (red bracket), cells of the developing vulva (cyan bracket), and intestinal cells near the tail of wild-type animals (cyan or red dashed lines for each pair of sister cells) are indicated. Scale bar = 10µm. Seam cells were silenced and not visible in wild-type animals. (**B**) Silencing by neuronal dsRNA in the absence of RRF-1 is most readily detected in intestinal cells and is highly variable. *Left*, Schematic showing regions where numbers of GFP-positive non-intestinal nuclei were counted (head, anterior to the posterior bulb of the pharynx; anterior body, anterior to the vulva; posterior body, posterior to the vulva). *Right*, Counts in non-intestinal cells included most

body-wall muscle and hypodermal cells throughout the animal but specifically excluded some other cell types. The excretory canal cell (detected in 0/10 *sur-5::gfp*, 10/10 *sid-1(-); sur-5::gf*p and 10/10 *rrf-1(-); sur-5::gfp* animals) and intestinal cells were excluded from region-specific analysis based on their large sizes and positions. Cells of the developing uterus and vulva, which remained visible in all tested strains, were excluded based on their small sizes and collective morphology. Seam cells, which show faint expression were excluded because they could not be reliably detected in most Z-slices that were away from the coverslip. Intestinal cells were counted separately. See Supplemental Movie S1 for an example of counting and Materials and Methods for thresholds used. Red bars indicate medians, asterisks and circle indicate p-value < 0.05 and p-value = 0.08, respectively (Student's t-test), and n = 10 L4-staged animals.

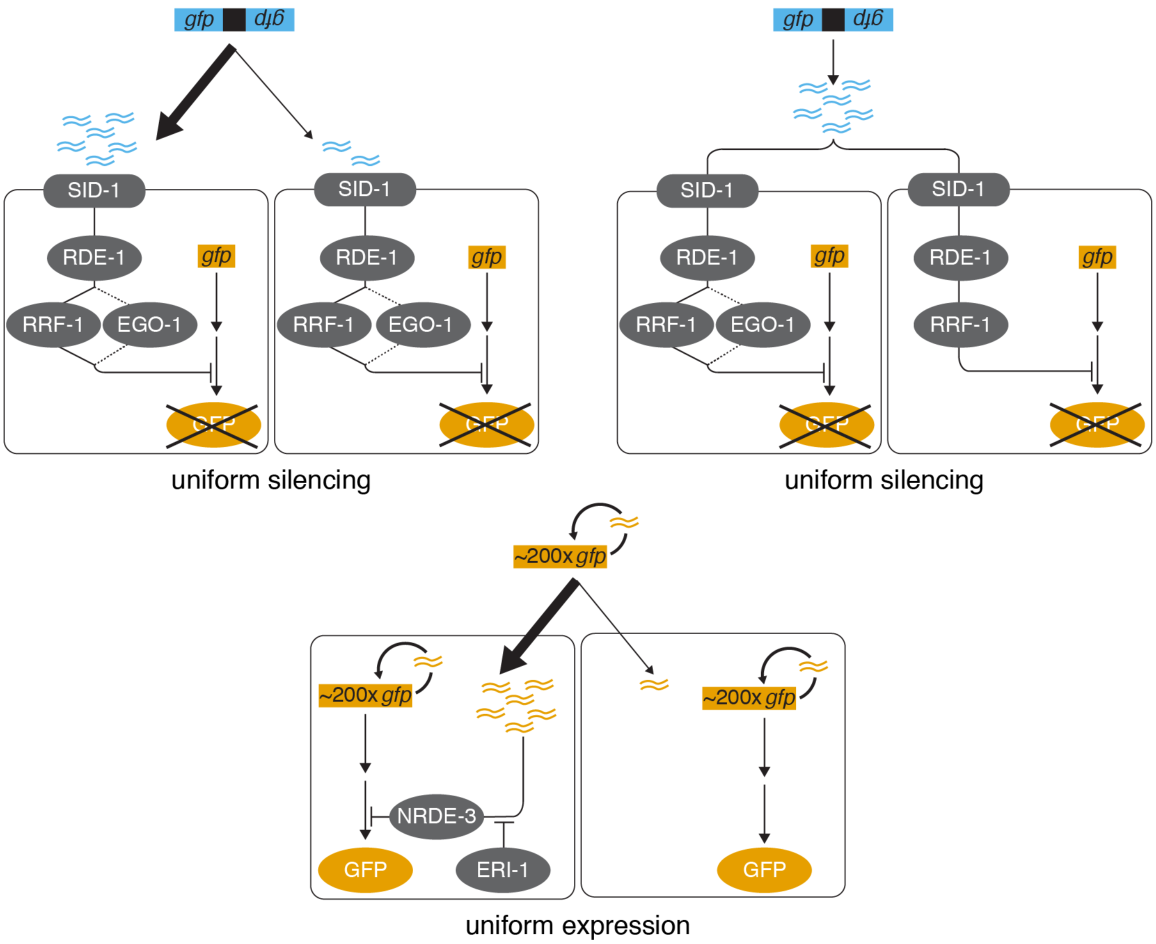

Supplementary Figure S9: Cells can appear similarly functional despite underlying differences. Two instances where homogeneity of outcome (uniform silencing or uniform gene expression) masks the variation in mechanisms are shown. *Top Left*, Equal availability of both RRF-1 and EGO-1 in all intestinal cells could underlie all silencing. Nevertheless, in the absence of RRF-1, each cell amplifies different amounts of small RNAs using EGO-1 because of the unequal and random availability of neuronal dsRNA. *Top Right*, Equal amounts of neuronal dsRNA can enter cells and engage either a single RdRP (RRF-1) or two RdRPs (RRF-1 and EGO-1) to eventually cause silencing. In the absence of RRF-1, the unequal and random availability of EGO-1 enables silencing only in some cells. Note that silencing enabled by EGO-1 could reflect either direct action in intestinal cells or indirect effects of its expression in the germline. In the presence of RRF-

1, this mosaicism is masked and uniform silencing occurs. *Bottom*, Double-stranded RNA derived from a multi-copy *gfp* transgene is segregated asymmetrically during early cell divisions (35). In the absence of ERI-1, the unequal and random availability of this dsRNA prevents expression only in some cells. In the presence of ERI-1, this mosaicism is masked and uniform expression occurs.

Supplementary Movie S1: Z-stack showing counted nuclei (flashes in white) in the anterior body (mostly body-wall muscle and hypodermal cells). See Materials and Methods for the rationale for including/excluding specific nuclei (uterine cells, intestinal cells, etc.).

SUPPLEMENTARY MATERIALS AND METHODS

Transgenesis and genome editing:

To express *rde-1(+)* under its own promoter (*Prde-1::rde-1(+)*): The *rde-1* promoter, coding sequence, and 3' UTR were amplified from N2 gDNA using the primers P17 and P18. AMJ233 animals were transformed with 10 ng/µl of *Prde-1::rde-1(+)* and 40ng/µl of pHC448 in dH_2O to generate three independent transgenic lines.

To express *rde-1(+)* in the neurons (*Prgef-1::rde-1(+)*): The *rgef-1* promoter (*Prgef-1*) was amplified using the primers P19 and P20, and *rde-1(+)* coding sequence and 3' UTR was amplified from N2 gDNA using the primers P21 and P22. The two PCR products were used as template and the *Prgef-1::rde-1(+)* fusion product was generated using the primers P23 and P24. AMJ233 animals were transformed with 10 ng/µl of *Prgef-1::rde-1(+)* and 40 ng/µl of pHC448 in dH_2O to generate three independent transgenic lines.

To express *rde-1(+)* in the intestine (*Psid-2::rde-1(+)*): The *sid-2* promoter (*Psid-2*) was amplified from N2 gDNA using the primers P25 and P26, and *rde-1(+)* coding sequence and 3' UTR was amplified from N2 gDNA using the primers P27 and P22. The two PCR products were used as template and the *Psid-2::rde-1(+)* fusion product was generated using the primers P28 and P24. AMJ233 animals were transformed with 10 ng/ul of *Psid-2::rde-1(+)* and 40 ng/µl of pHC448 in dH_2O to generate three independent transgenic lines.

To express *rrf-1* in most somatic cells (*Psur-5::rrf-1(+)*): The precise promoter elements that drive *rrf-1* expression are unclear because *rrf-1* is the downstream gene in an operon that includes another RNA-dependent RNA polymerase gene *ego-1*. Therefore, we used the promoter of a somatically expressed gene *sur-5* to express *rrf-1* in most somatic cells. The *sur-5* promoter was amplified from N2 gDNA using the primers P29 and P30. The *rrf-1* gene and its 3'UTR were amplified together from N2 gDNA using the primers P31 and P32. The two PCR products were used as templates to generate the fusion product using

the primers P33 and P34. A 1:4 mixture of *Psur-5::rrf-1(+)* (10 ng/µl) and pHC448 (40 ng/µl) in 10mM Tris HCl (pH 8.5) was injected into AMJ241 to generate AMJ294 and two other independent transgenic lines.

To express *rrf-1* in intestinal cells (*Psid-2::rrf-1(+)*): The *sid-2* promoter was amplified from N2 gDNA using the primers P35 and P36, and the *rrf-1* coding sequence was amplified along with its 3' UTR using the primers P31 and P32. The two PCR products were used as templates to generate the fusion product using the primers P35 and P34. A 1:4 mixture of *Psid-2::rrf-1(+)* (10 ng/µl) and pHC448 (40 ng/µl) in 10mM Tris HCl (pH 8.5) was injected into AMJ241 to generate AMJ296 and two other independent transgenic lines.

To express *rrf-1* in the neurons (*Prgef-1::rrf-1(+)*): The *rgef-1* promoter was amplified from N2 gDNA using the primers P37 and P38 and the *rrf-1* coding sequence and its 3'UTR were amplified together using the primers P31 and P32. The two PCR products were used as templates to generate the fusion product using the primers P37 and P34. A 1:4 mixture of *Prgef-1::rrf-1(+)* (10 ng/µl) and pHC448 (40 ng/µl) in 10mM Tris HCl (pH 8.5) was injected into AMJ241 to generate AMJ295 and two other independent transgenic lines.

To delete *rrf-2* using genome editing: The forward primers P39 and P40 were used to amplify two guide RNAs for the *rrf-2* deletion (Supplementary Figure S4) and the forward primer P41 was used to amplify the guide RNA for the co-conversion marker *dpy-10*. The homology repair templates for *rrf-2* and *dpy-10* were single-stranded DNA oligos (P42 for *rrf-2* and P43 for *dpy-10*). AMJ349 (*Peft-3::gfp; qtIs49*) animals were injected with 5.1 pmol/µl of *rrf-2* guide RNA1, 6.2 pmol/µl of *rrf-2* guide RNA2, 2.3 pmol/µl of *dpy-10* guide RNA, 9.4 pmol/µl of *rrf-2* homology repair template, 6.1 pmol/µl of *dpy-10* homology repair template and 1.5 pmol/µl of Cas9 protein (PNA Bio Inc.). The deletion was genotyped using 3 primers (P44-P46) and one strain with a homozygous allele was designated as AMJ979.

To delete *rrf-3* using genome editing: Two crRNAs were combined with tracrRNA to create an *rrf-3* deletion (Supplementary Figure S4) using a single-stranded DNA homology repair template. AMJ973 animals were injected with 5.0 pmol/μl of *rrf-3* crRNA1(P47) and *rrf-3* guide crRNA2 (P48), 7.2 pmol/μl of tracrRNA (P49), 2.5 pmol/μl of *dpy-10* crRNA (P50), 9.4 pmol/μl of *rrf-3* homology repair template (P51), 9.3 pmol/μl of *dpy-10* homology repair template (P43) and 0.3 pmol/μl of Cas9 protein (IDT). The deletion was genotyped using 3 primers (P52-P54) and one strain with a homozygous allele was designated as AMJ1252.

To mutate *mut-16, rde-11* and *ego-1* using genome editing: A single crRNA was combined with tracrRNA to target a region of *mut-16*, *rde-11* or *ego-1.* Mutations in *mut-16* were created in AMJ973 and AMJ977. Mutations in *rde-11* were created in HC195, HC567 and AMJ301. Mutations in *ego-1* were created in AMJ973, AMJ976 and AMJ977. Animals were injected with 5 pmol/μl of crRNA (P55 for *mut-16,* P56 for *rde-11* and P57 for *ego-1)*, 4.5-7.2 pmol/μl of tracrRNA, 2.5 pmol/μl of *dpy-10* crRNA (P50), ~9 pmol/μl of homology repair template (P58 for *mut-16*, P59 for *rde-11* and P60 for *ego-1*), 9.3 pmol/μl of *dpy-10* homology repair template (P43) and 0.3 pmol/μl of Cas9 protein (IDT). Mutations were genotyped using either the elimination of a restriction site by the point mutation (e.g *rde-11*, genotyped using P05-P06, restriction enzyme: PvuII, strains with a homozygous allele in different backgrounds designated as AMJ1264, AMJ1304 and AMJ1305), or by the addition of silent point mutations that eliminate a nearby restriction site (e.g *mut-16*, genotyped using P14-P15, restriction enzyme: PstI, strains with a homozygous allele in different backgrounds designated as AMJ974 and AMJ983, and *ego-1*, genotyped using P61-P62, restriction enzyme: BstBI, strains with a homozygous allele in different backgrounds designated as AMJ1262, AMJ1263 and AMJ1299). Sanger sequencing revealed that the mutation created in *ego-1* in AMJ1262 is an insertion/deletion that

disrupts the reading frame (Q171fs, resulting in a stop codon 13 amino acids later, sequencing by Maïgane Diop, Jose lab).

To tag *gtbp-1*, *unc-22* and *sur-5* with *gfp* using genome editing: A single guide RNA was selected <6 base pairs away from the insertion site (Supplementary Figure S4), and *gfp* sequences for the homology template was amplified from pTK2 (a derivative of pPD95.75 – Addgene plasmid #1494, a gift from Andrew Fire) using primers with 35-40 base pair overhangs matching either side of the cut site. Guide RNAs were amplified using the forward primers P63 for *gtbp-1::gfp*, P64 for *gfp::unc-22*, P65 for *unc-22::gfp* and P66 for *sur-5::gfp*. Homology templates were amplified using P67 and P68 for *gtbp-1::gfp*, P69 and P70 for *gfp::unc-22*, P71 and P72 for *unc-22::gfp*, and P73 and P74 for *sur-5::gfp*. N2 animals were injected with 9-15 pmol/μl of guide RNA, 0.4 to 0.9 pmol/μl of homology repair template and 1.5 pmol/μl of Cas9 protein (PNA Bio Inc.). Edited F1 or F2 animals were selected by picking animals that showed GFP fluorescence under the Olympus MVX10 fluorescent microscope. The GFP insertion was genotyped using 3 primers (P75 within GFP along with P76 and P77 for *gfp::unc-22*, or P78 and P79 for *unc-22::gfp,* or P80 and P81 for *sur-5::gfp*). Strains with homozygous alleles were designated as AMJ1000 for *gfp::unc-22,* AMJ1001 for *unc-22::gfp* and AMJ975 for *sur-5::gfp***.**

Table S1. Strains used.

**Strains**	**Genotype**
N2	Wild type
AMJ1	*qtIs50 [Pmyo-3::DsRed2, Pmyo-3::DsRed2*-dsRNA *& sid-1(+)] II; juIs73 [Punc-25::gfp] qtIs49 [Prgef-1::gfp*-dsRNA *& pRF4] III; eri-1(mg366) nrIs20 [sur-5::gfp] IV*
AMJ2	*qtIs49 III; eri-1(mg366) nrIs20 IV; sid-1(qt9) V*
AMJ38	*rrf-1(ok589) I; qtEx136 [Prgef-1::unc-22*-dsRNA*]*
AMJ86	*rrf-1(jam2) I; qtIs50 II; juIs73 qtIs49 III; eri-1(mg366) nrIs20 IV*
AMJ87	*rrf-1(jam3) I; qtIs50 II; juIs73 qtIs49 III; eri-1(mg366) nrIs20 IV*
AMJ109	*rrf-1(jam4) I; qtIs50 II; juIs73 qtIs49 III; eri-1(mg366) nrIs20 IV*
AMJ113	*qtIs50 II; juIs73 qtIs49 III; eri-1(mg366) nrIs20 IV; rde-1(jam1) V*
AMJ116	*qtIs50 II; juIs73 qtIs49 III; eri-1(mg366) nrIs20 IV; rde-11(jam51) V*
AMJ119	*qtIs50 II; juIs73 qtIs49 III; eri-1(mg366) nrIs20 IV; rde-11(jam50) V*
AMJ120	*qtIs50 II; juIs73 qtIs49 III; eri-1(mg366) nrIs20 IV; sid-1(jam52) V*
AMJ233	*qtIs50 II; juIs73 qtIs49 III; eri-1(mg366) nrIs20 IV; rde-1(jam1) V [1x outcross]*
AMJ241	*rrf-1(jam3) I; qtIs50 II; juIs73 qtIs49 III; eri-1(mg366) nrIs20 IV [1x*
AMJ245	*rrf-1(ok589) I; qtIs49 III; eri-1(mg366) nrIs20 IV*
AMJ246	*rrf-1(ok589) I; nrIs20 eri-1(mg366) IV*
AMJ247	*rrf-1(ok589) I; qtIs49 III; nrIs20 IV*
AMJ248	*rrf-1(ok589) I; nrIs20 IV*
AMJ294	*rrf-1(jam3) I; qtIs49 III; eri-1(mg366) nrIs20 IV; jamEx71 [Psur-5::rrf-1(+)]*
AMJ295	*rrf-1(jam3) I; qtIs49 III; eri-1(mg366) nrIs20 IV; jamEx72 [Prgef-1::rrf-1(+)]*
AMJ296	*rrf-1(jam3) I; qtIs49 III; eri-1(mg366) nrIs20 IV; jamEx73 [Psid-2::rrf-1(+)]*
AMJ300	*qtIs49 III; nrIs20 IV*
AMJ318	*qtIs49 III; eri-1(mg366) nrIs20 IV; rde-1(ne219) V*
AMJ349	*qtIs49 III; oxSi221 [Peft-3::gfp] II; unc-119(ed3)? III*
AMJ477	*qtEx136*
AMJ793	*jamEx203 [Prgef-1::bli-1*-dsRNA*]*
AMJ964	*rrf-1(ok589) I; jamEx203*
AMJ965	*unc-22(jam77[gfp::unc-22]) IV; qtEx136*
AMJ966	*rrf-1(ok589) I; unc-22(jam77[gfp::unc-22]) IV; qtEx136*
AMJ967	*unc-22(jam77[gfp::unc-22]) IV; qtEx140 [Prgef-1::gfp*-dsRNA*]*
AMJ968	*rrf-1(ok589) I; unc-22(jam77[gfp::unc-22]) IV; qtEx140*
AMJ969	*unc-22(jam78[unc-22::gfp]) IV; qtEx136*
AMJ970	*rrf-1(ok589) I; unc-22(jam78[unc-22::gfp]) IV; qtEx136*
AMJ971	*unc-22(jam78[unc-22::gfp]) IV; jamEx140*
AMJ972	*rrf-1(ok589) I; unc-22(jam78[unc-22::gfp]) IV; jamEx140*
AMJ973	*rrf-1(ok589) I; oxSi221 II; qtIs49 unc-119(ed3)? III*
AMJ974	*rrf-1(ok589) mut-16(jam91) I; oxSi221 II; qtIs49 unc-119(ed3)? III*
AMJ975	*sur-5(jam79[sur-5::gfp]) IV*
AMJ976	*qtIs49 III; sur-5(jam79[sur-5::gfp]) IV*
AMJ977	*rrf-1(ok589) I; qtIs49 III; sur-5(jam79[sur-5::gfp]) IV*
AMJ978	*qtIs49 III; sur-5(jam79[sur-5::gfp]) IV; sid-1(qt9) V*

AMJ979	*rrf-2(jam36[deletion]) I; oxSi221 II; qtIs49 unc-119(ed3)? III*
AMJ980	*rrf-1(ok589) rrf-2(jam36[deletion]) I; oxSi221 II; qtIs49 unc-119(ed3)? III*
AMJ982	*eri-1(mg366) sur-5(jam79) IV*
AMJ983	*rrf-1(ok589) mut-16(jam92) I; qtIs49 unc-119(ed3)? III; sur-5(jam79[sur-5::gfp]) IV*
AMJ984	*qtIs49 III; unc-22(jam77[gfp::unc-22])IV*
AMJ985	*rrf-1(ok589) I; qtIs49 III; unc-22(jam77[gfp::unc-22])IV*
AMJ986	*qtIs49 unc-119(ed3)? III; oxSi346 [Peft-3::gfp] IV*
AMJ987	*rrf-1(ok589) I; qtIs49 unc-119(ed3)? III; oxSi346 IV*
AMJ988	*qtIs49 unc-119(ed3)? III; oxSi230 [Peft-3::gfp] X*
AMJ989	*rrf-1(ok589) I; qtIs49 unc-119(ed3)? III; oxSi230 X*
AMJ990	*oxSi257 [Peft-3::gfp] I; qtIs49 unc-119(ed3)? III.*
AMJ991	*rrf-1(ok589) I; oxSi257 I; qtIs49 unc-119(ed3)? III.*
AMJ1000	*unc-22(jam77[gfp::unc-22]) IV*
AMJ1001	*unc-22(jam78[unc-22::gfp]) IV*
AMJ1163	*oxSi221 II; qtIs49 unc-119(ed3)? III; rde-11(hj37) IV*
AMJ1177	*mut-2(jam9) I; oxSi221 II; qtIs49 unc-119(ed3)? III*
AMJ1179	*mut-16(pk710) I; oxSi221 II; qtIs49 unc-119(ed3)? III*
AMJ1180	*qtIs49 III; gtbp-1(ax2053[gtbp-1::gfp])IV*
AMJ1181	*rrf-1(ok589) I; qtIs49 III; gtbp-1(ax2053[gtbp-1::gfp])IV*
AMJ1183	*qtIs49 III; gtbp-1(jam83[gtbp-1::gfp])IV*
AMJ1184	*rrf-1(ok589) I; qtIs49 III; gtbp-1(jam83[gtbp-1::gfp])IV*
AMJ1252	*rrf-1(ok589); rrf-3(jam95[deletion]) oxSi221 II; qtIs49 unc-119(ed3)? III*
AMJ1262	*rrf-1(ok589) ego-1(jam93) I; oxSi221 II; qtIs49 unc-119(ed3)? III*
AMJ1263	*ego-1(jam94) I; qtIs49 III; sur-5(jam79[sur-5::gfp]) IV*
AMJ1264	*nrIs20 rde-11(jam96) IV*
AMJ1299	*rrf-1(ok589) ego-1(jam93) I; qtIs49 III; sur-5(jam79[sur-5::gfp]) IV*
AMJ1304	*eri-1(mg366) nrIs20 rde-11(jam104) IV*
AMJ1305	*qtIs49 unc-119(ed3)? III; nrIs20 rde-11(jam105) IV*
EG6070	*oxSi221 II; unc-119(ed3) III.*
EG6109	*unc-119(ed3) III; oxSi230 X.*
EG6171	*oxSi257 I; unc-119(ed3) III.*
EG6401	*unc-119(ed3) III; oxSi346 IV*
HC196	*sid-1(qt9) V*
HC567	*eri-1(mg366) nrIs20 IV*
HC780	*rrf-1(ok589) I [2x]*
JH3197	*gtbp-1(ax2053[gtbp-1::gfp])IV*
VS27	*rde-11(hj37) IV*
WM27	*rde-1(ne219) V*

Table S2. Oligonucleotides used.

Name	Sequence
P01	cagacctcacgatatgtggaaa
P02	ggaacatatggggcattcg
P03	cggacagaggaagaaatgc
P04	cactattcacaagcattggc
P05	gatttcggactccctatgtg
P06	agttaatgtagcacccgactc
P07	agtaacagtttcaaatggccg
P08	tcttcactgtacaatgtgacg
P09	tgccatcgcagatagtcc
P10	tggaagcagctaggaacag
P11	ccgtgacaacagacattcaatc
P12	catttgtgcatttccttcca
P13	atgcttgtgaaatccgggta
P14	ttctggatactcctcggatg
P15	ttatttcgagtcgttcagagc
P16	aaaagcaccgactcggt
P17	cggagtgagtaccaatgagc
P18	gggacgaagtattgcggag
P19	tttccgatacccccttatatc
P20	aaaattcgaggacatgtcgtcgatgccgtcttc
P21	gaagacggcatcgacgacatgtcctcgaattttcccg
P22	ccggagtgagtaccaatgag
P23	gatacccccttatatcagcac
P24	gagcgatgtcatcttgtgacc
P25	ctcattttcgggttcagtgg
P26	aaaattcgaggacatttcctgaaaatatcagggttttg
P27	caaaaccctgatattttcaggaaatgtcctcgaattttcccg
P28	gttcagtggtttgtcaactc
P29	agtatcggaattgagatggg
P30	ccatgacttcgttccgacattctgaaaacaaaatgtaaagttc
P31	atgtcggaacgaagtcatgg
P32	agacacactcttcagcgaac
P33	gaaattgaagacgcaacaaaaac
P34	cgaaaagagaacggagtgtc
P35	ctcattttcgggttcagtgg
P36	ccatgacttcgttccgacatttcctgaaaatatcagggttttg
P37	cgataatctcgtgacactcg
P38	ccatgacttcgttccgacatcgtcgtcgtcgtcgatgc
P39	atttaggtgacactatagcaggcctttcgttatgtcggttttagagctagaaatagcaag
P40	atttaggtgacactatagtctggaaattgcactctgggttttagagctagaaatagcaag
P41	atttaggtgacactatagctaccataggcaccacgaggttttagagctagaaatagcaag

P42	ggtgaaattacttctcgttttgcaggcctttcgtttggtgggctacgttttgatagtcatgtcacgaaat
P43	cacttgaacttcaatacggcaagatgagaatgactggaaaccgtaccgcatgcggtgcctatggtagcg gagcttcacatggcttcagaccaacagccta
P44	ttagcgtctcttagctgtctg
P45	ctaaacttacaagcccatagg
P46	cgcaattctcgtagatcaaac
P47	ctagagtcatcattatcaaa
P48	aacataaaacatgatataag
P49	agcauagcaaguuaaaauaaggcuaguccguuaucaacuugaaaaaguggcaccgagucgg ugcuuu
P50	gctaccataggcaccacgag
P51	ttttcaaaaagtctttcacaaaactgctcccgtttatatcatgttttatgttttgcttgttttatagaat
P52	tcaatggagcgaacatacatc
P53	ctgtagcaggtaatcacgaag
P54	taataactccgatctcgatgc
P55	atgcacttgtacgaggaggt
P56	tcatttttcttcagctgga
P57	ggaatgaaccaaatcaaag
P58	ttcagaccgaacacgcccatgcacttgtacgaggaggttagcttcagtccaaagagggcgcattctttcc
P59	aaaatgtctagtttaaaattcatttttcttcagctagacggactgtatctctacaaaaaacacgacgatg
P60	aacacaagccgataaaattggaatgaactaaatcaaagtcgagtttgaattcgataaaattgattttatg
P61	aattatgaagttgcgaccagc
P62	gttcggtaggctcttctaatg
P63	atttaggtgacactatagcacgaggtggtatgcgcaggttttagagctagaaatagcaag
P64	atttaggtgacactatagaagcgcggtgcgccaaccagttttagagctagaaatagcaag
P65	atttaggtgacactataggtggcaggatgattagacagttttagagctagaaatagcaag
P66	atttaggtgacactatagatctttaattttatttcaagttttagagctagaaatagcaag
P67	ggttcgggtggtgctccacgaggtggtatgcgcatgagtaaaggagaagaacttttc
P68	cttctaattttgtcccgcattttggaaaccgcttttgtatagttcatccatgcc
P69	attaaaagtcgtgcgccggcccctccaacttcgaccatgagtaaaggagaagaacttttc
P70	aatggacggtttctgggtgaagcgcggtgcgccaaccattttgtatagttcatccatgcc
P71	gcaattgcaaaatatgcggcagctcttctccttgtcatgagtaaaggagaagaacttttc
P72	aaatataatgaagtttaaagtggtggtggcaggatgactatttgtatagttcatccatgc
P73	ccagaatctcttgatcatttcgttcaatacagacttatgagtaaaggagaagaacttttc
P74	gaaggaaagcgtgaatatataattttcagaaacagaaacaaataaatcgttgaaataaaattaaagact atttgtatagttcatccatgc
P75	gtgtccaagaatgtttccatc
P76	cgtcatagaagagacagtttg
P77	gaacagagataggtgagatatg
P78	gaatagttgtggatacgctag
P79	ccgggcttattacttgattg
P80	cagccattgtttctaaactcc
P81	aacaggttcaggcaatgagc

Table S3. Sequencing statistics.

mutant	mapped reads (number)	mapped reads (%)	read length (bases)	genome covered (%)	depth of coverage (fold)
rrf-1(jam2)	38,474,029	82.8	97	99.92	37.3
rrf-1(jam3)	38,862,516	86.8	97	99.96	37.7
rrf-1(jam4)	34,096,083	84.0	97	99.96	33.1
rde-1(jam1)	30,401,686	81.7	97	99.96	29.5
rde-11(jam50)	42,053,854	84.4	97	99.97	40.8
rde-11(jam51)	38,988,725	84.0	97	99.97	37.8
sid-1(jam52)	26,823,782	73.7	97	99.95	26.0
